# The Pathophysiological Role of CoA

**DOI:** 10.3390/ijms21239057

**Published:** 2020-11-28

**Authors:** Aleksandra Czumaj, Sylwia Szrok-Jurga, Areta Hebanowska, Jacek Turyn, Julian Swierczynski, Tomasz Sledzinski, Ewa Stelmanska

**Affiliations:** 1 Department of Pharmaceutical Biochemistry, Faculty of Pharmacy, Medical University of Gdansk, 80-211 Gdańsk, Poland; Aleksandra.czumaj@gumed.edu.pl; 2 Department of Biochemistry, Faculty of Medicine, Medical University of Gdansk, 80-211 Gdansk, Poland; szrok@gumed.edu.pl (S.S.-J.); areta.hebanowska@gumed.edu.pl (A.H.); jacek.turyn@gumed.edu.pl (J.T.); 3 State School of Higher Vocational Education in Koszalin, 75-582 Koszalin, Poland; juls@gumed.edu.pl

**Keywords:** CoA, coenzyme A, CoAlation, cancer, neurodegenerative diseases

## Abstract

The importance of coenzyme A (CoA) as a carrier of acyl residues in cell metabolism is well understood. Coenzyme A participates in more than 100 different catabolic and anabolic reactions, including those involved in the metabolism of lipids, carbohydrates, proteins, ethanol, bile acids, and xenobiotics. However, much less is known about the importance of the concentration of this cofactor in various cell compartments and the role of altered CoA concentration in various pathologies. Despite continuous research on these issues, the molecular mechanisms in the regulation of the intracellular level of CoA under pathological conditions are still not well understood. This review summarizes the current knowledge of (a) CoA subcellular concentrations; (b) the roles of CoA synthesis and degradation processes; and (c) protein modification by reversible CoA binding to proteins (CoAlation). Particular attention is paid to (a) the roles of changes in the level of CoA under pathological conditions, such as in neurodegenerative diseases, cancer, myopathies, and infectious diseases; and (b) the beneficial effect of CoA and pantethine (which like CoA is finally converted to Pan and cysteamine), used at pharmacological doses for the treatment of hyperlipidemia.

## 1. Introduction

Coenzyme A (CoA or CoA-SH) is an essential cofactor of cellular metabolism in all living organisms. Pantothenic acid (Pan, commonly known as vitamin B5) is the only nutritionally essential component involved in the synthesis of CoA, which is required for many biochemical processes (see below) and for the synthesis of an acyl carrier protein that is involved in fatty acid biosynthesis [1,2]. Moreover, Pan triggers immune cells to produce cytokines [3].

The name of Pan is derived from the root word *pantos*, which means “everywhere.” The widespread availability of Pan in the diet (both in products of animal, including milk, and plant origin) means that, in humans, Pan deficiency occurs largely due to severe malnutrition with combined vitamin deficiencies. However, it was recently found that cerebral Pan levels are significantly decreased compared to the control values in patients with Huntington’s disease [4]. Moreover, Pan deficiency results in greying hair in rats and other animals. Studies have shown that vitamin B_5_ supplementation with calcium pantothenate can promote grey hair darkening [5]. Neither the toxicity nor the upper intake level have been established [6]. However, in some individuals consuming very large doses of Pan supplements (approximately 10 g per day), gastrointestinal distress and diarrhea have been observed [2].

The unique chemical structure of CoA-SH allows it to be used to activate carboxylic acids involved in both catabolic and anabolic reactions. Generally, in humans, CoA-SH is required for (a) chemical reactions that generate energy from fat, carbohydrates, protein and catabolism of ethanol; (b) biosynthesis of fatty acids (necessary for biosynthesis of: triacylglycerols, phospholipids, sphingolipids), cholesterol, acetylcholine, prenyl moieties, bile acids, ketone bodies, heme, melatonin, glycosaminoglycans, glycoproteins, gangliosides, proteoglycans, and others) [7,8]; (c) regulation of metabolism (direct allosteric regulation of pyruvate dehydrogenase kinase-PDK, carnitine palmitoyltransferase 1—CPT1 and indirect regulation of carbamoyl phosphate syntethase I); and (d) gene expression (e.g., histone acetylation) [9]. Moreover, CoA-SH and its thioester derivatives (mainly acetyl-CoA and benzoyl-CoA) participate in detoxification reactions during which compounds are formed and then excreted in urine, e.g., hippuric or mercapturic acids [10]. The recently discovered, unconventional function of free CoA (CoA-SH) is protein CoAlation. This process is related to redox regulation and antioxidant defense [11]. Exemplary reactions that involve CoA-SH as a substrate and reactions in which CoA-SH is released as a product in human cells are presented in Table 1 and Table 2.

The major pools of CoA-SH and its thioesters are found in mitochondria and the cytosol. Other organelles (peroxisome, nuclei, lysosomes, and endoplasmic reticulum) contain much less CoA-SH. In mitochondria, CoA-SH is used in: (a) fatty acids and ketone bodies oxidation (as a substrate for thiolases and carnitine palmitoyltransferase 2—CPT2); (b) tricarboxylic acid cycle (as a substrate for α-oxoglutarate dehydrogenase); and (c) oxidative decarboxylation of pyruvate and branched-chain α-keto acids [12]. In the cytosol, CoA-SH is mainly used in reactions catalyzed by ATP-citrate lyase (ACLY) and acyl-CoA synthetase (ACS) (Table 1).

**Figure 1:**
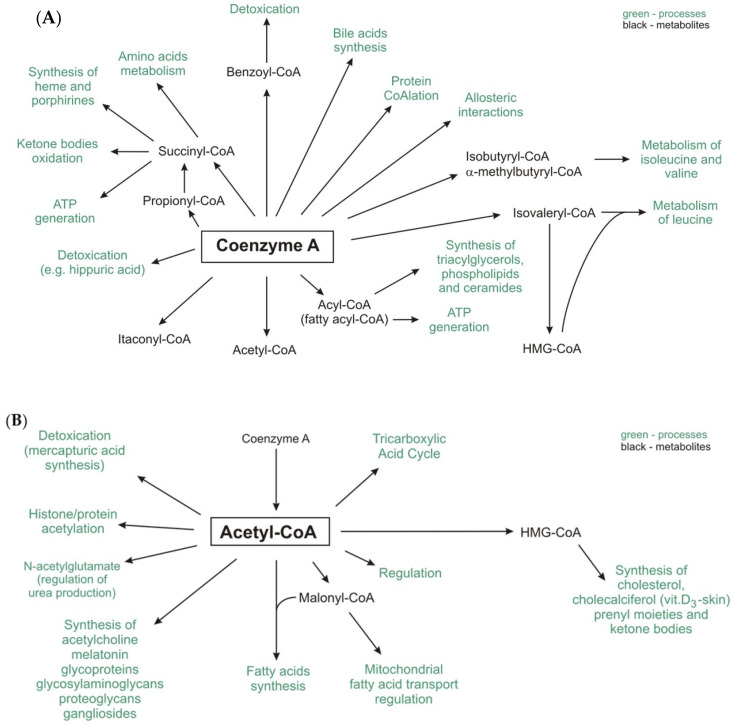
The role of CoA-SH (**A**) and acetyl-CoA (**B**) in human metabolism.

The acyl groups formed during the metabolism of glucose, amino acids and fatty acids in human cells and those produced by gut microbiota are attached to CoA-SH to form its thioester derivatives, such as acetyl-CoA, succinyl-CoA, propionyl-CoA, isovaleryl-CoA, isobutyryl-CoA, α-methylbutyryl-CoA, and fatty acyl-CoA (commonly referred to as acyl-CoA), e.g., palmitoyl-, oleoyl-, and stearoyl-CoA (Figure 1A).

Itaconyl-CoA, a derivative of a newly discovered mammalian metabolite, itaconate, inhibits B12-dependent methylmalonyl-CoA mutase [14].

Among the abovementioned compounds, acetyl-CoA is the central and most important metabolite and forms an intersection between the anabolic and catabolic pathways [15]. Moreover, other important metabolites, such as malonyl-CoA (a substrate of lipogenesis and regulator of fatty acid oxidation) and 3-hydroxy-3-methylglutaryl-CoA (HMG-CoA) (a substrate for cholesterol and ketone bodies synthesis), are formed from acetyl-CoA (Figure 1B) [15,16].

The pool of CoA-SH in the cell is replenished by the enzymes that release it from thioester compounds, e.g., citrate synthase, acyl-coenzyme A: cholesterol acyltransferase (ACAT), many acyl- and acetyltransferases, acyl-CoA thioesterases, fatty acid synthase (FASN), fatty acid elongase (ELOVL), 3-hydroxy-3-methylglutaryl-CoA reductase (HMGR), and CPT1 (Table 2), [17,18,19,20,21].Notably, changes in the CoA-SH/acetyl-CoA ratio affect not only the regulation of energy metabolism but also the regulation of other cellular processes, such as autophagy, mitosis, and cell death [20,22].

This review summarizes current knowledge of CoA-SH subcellular concentrations, the roles of CoA-SH synthesis and degradation processes and changes in the level of CoA-SH under pathological conditions, such as neurodegenerative diseases, cancer, myopathies, infectious diseases and the genetic make-up of CoA-SH genes. Finally, the beneficial effects of CoA-SH and pantethine (a dimer of the CoA precursor pantetheine) used at pharmacological doses for the treatment of hyperlipidemia are presented.

## 2. CoA Synthesis and Degradation in Mammalian Cells

### 2.1. CoA Synthesis

The substrates for CoA-SH biosynthesis are Pan, cysteine and ATP. There are three main sources of blood-circulating Pan: (a) food; (b) gut microbiota; and (c) extracellular products of CoA-SH degradation of damaged cells and recycled cellular content (exocytosis) [23,24,25,26]. In the diet, CoA-SH is the main source of Pan (approx. 85% of dietary Pan is in the form of CoA-SH or phosphopantetheine), which is hydrolyzed in the intestine, and Pan, which is thus formed, is then absorbed [26,27,28]. In mammals, Pan is absorbed in the intestine and transported into cells through sodium-dependent multivitamin transporter (SMVT) [23,27]. Pan delivered into the bloodstream is transported throughout the body (to organs) by red blood cells [29].

**Figure 2:**
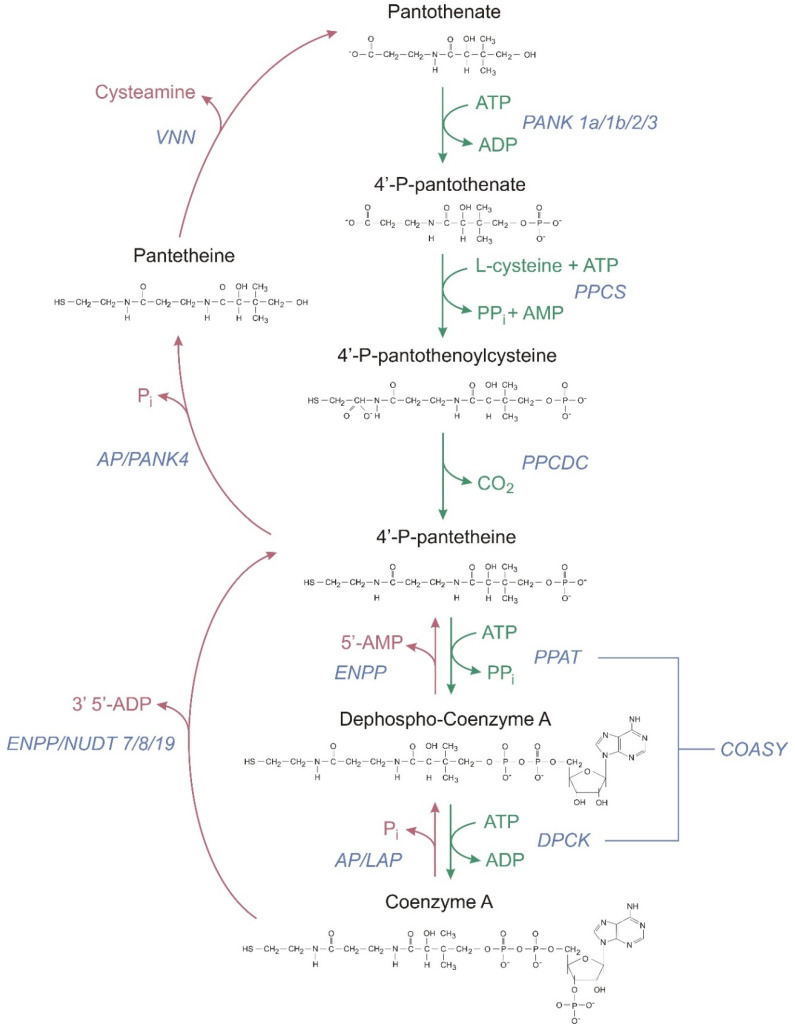
Coenzyme A biosynthetic and degradation pathways in humans. Green arrows indicate CoA synthesis and red arrows indicate CoA degradation. PANK—pantothenate kinase, PPanSH—4′-phosphopantetheine, PPCS—phosphopantothenoylcysteine synthetase, PPCDC—phosphopantothenoylcysteine decarboxylase, COASY—CoA synthase, PPAT—4′-phosphopantetheine adenyltransferase, DPCK—dephospho-CoA kinase, ENPP—ectonucleotide pyrophosphatase/phosphodiesterase, AP—alkaline phosphatase, VNN—pantetheinase, NUDT—intracellular degradation, nudix (nucleoside diphosphate linked moiety X)-type motif, and LAP—lysosomal acid phosphatase.

The first step of CoA-SH biosynthesis is the phosphorylation of intercellular Pan to 4′-phosphopantothenate (PPan) by pantothenate kinase (PANK). Then PPan is condensed with cysteine by phosphopantothenoylcysteine synthetase (PPCS), followed by decarboxylation by phosphopantothenoylcysteine decarboxylase (PPCDC) to generate 4′-phosphopantetheine (PPanSH) [30]. Human PPCS utilizes ATP to activate substrate in a ligation reaction. An acyl adenylate intermediate is formed, followed by release of pyrophosphate, and binding of cysteine, and then, the final products, 4′-phosphopantothenoylcysteine and AMP, are formed [31]. In mammals, in contrast to other organisms, the last two steps in the CoA-SH biosynthetic pathway are catalysed by CoA synthase (COASY), which exhibits two enzymatic activities: 4′-phosphopantetheine adenyltransferase (PPAT) and a dephospho-CoA kinase (DPCK) [30]. PPanSH is first converted to dephospho-CoA (dPCoA) and subsequently phosphorylated to form CoA-SH. The CoA-SH synthesis pathway is presented in Figure 2.

Regulation of CoA-SH synthesis takes place mainly at the level of the first reaction (PANK) [32]. There are four active mammalian (human) PANK isoforms involved in this process (PANK1α, PANK1β, PANK2, and PANK3) encoded by three distinct genes (*PANK1-3*). They are localized in different subcellular compartments, which allows them to acts as local sensors of CoA levels and regulate the biosynthetic pathway [33]. Human PANK proteins share a homologous C-terminal catalytic domain, but they have different N-termini. PANK1α is exclusively localized in the nucleus, whereas PANK1β is localized in the cytosol and is association with clathrin-coated vesicles and recycling endosomes [34]. Human PANK2 is detected in the nucleus and mitochondria, specifically in the intermembrane space. The PANK3 isoform is exclusively localized in the cytosol [34]. The different subcellular localizations of the PANK isoforms enable these proteins to act as metabolic sensors that modulate the rate of CoA-SH synthesis to align with the cellular metabolic state. The reactions catalyzed by PANKs are rate-limiting and subjected to feedback regulation by CoA-SH or its derivatives (mainly acetyl-CoA). It is thought that PANK2 and PANK3 are not active under physiological conditions associated with high acetyl-CoA levels, especially in mitochondria [33]. The different isoforms most likely compensate for each other to maintain adequate CoA-SH levels.

COASY is a secondary regulatory site, which become important only when the flux through the pathway is dramatically increased by overexpression of PANK [32]. In mammalian cells, there is one gene for COASY and it encodes three isoforms. Activities of mammalian COASY were reported in the cytosol, the outer mitochondrial membrane and the mitochondrial matrix, and it is probably anchored to the inner mitochondrial membrane [30,35]. Mitochondrial COASY is activated by phospholipids [35]. It has been proposed that COASY may function as a scaffold protein for the formation of the CoA-SH biosynthetic complex [30]. COASY activity is regulated by the phosphorylation/dephosphorylation of tyrosine residues. This posttranslational modification affects the interactions of COASY with the SH2 domain in several signaling proteins. Fasting, glucagon, glucocorticoids, oxidative stress and treatment with hypolipidemic drugs can increase the total level of CoA-SH. On the other hand, glucose, insulin, fatty acids and pyruvate were shown to decrease the level of intracellular CoA-SH [30,36].

**Figure 3:**
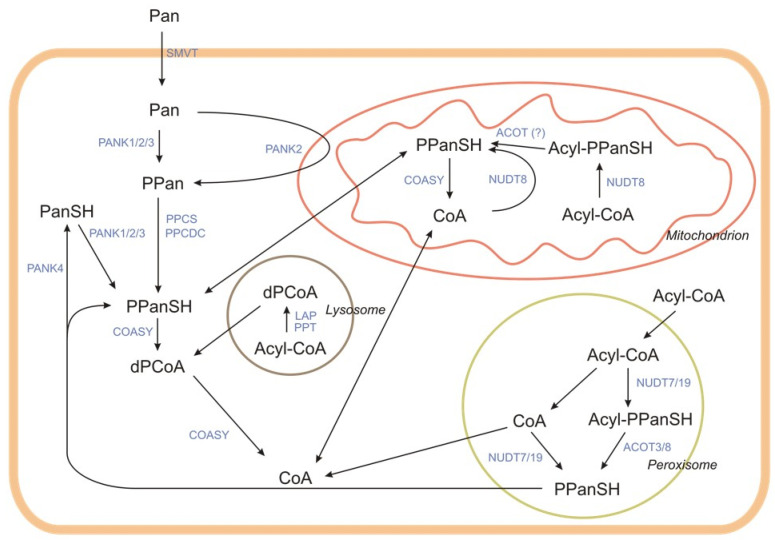
The compartmentalization and interplay of intracellular CoA synthesis and degradation pathways. Pan—pantothenate, PPan—4′-phosphopantothenate, PPanSH—4′-phosphopantetheine, dPCoA—dephospho-CoA, PANK—pantothenate kinase, PPCS—phosphopantothenoylcysteine synthetase, PPCDC—phosphopantothenoylcysteine decarboxylase, COASY—CoA synthase, PanSH—pantetheine, NUDT—intracellular degradation, nudix (nucleoside diphosphate linked moiety X)-type motif, LAP—lysosomal acid phosphatase, SMVT—sodium-dependent multivitamin transporter, and ACOT—acyl-CoA thioesterase. The figure was made based on Naquet et al. [23].

The enzymes involved in CoA-SH synthesis and degradation are localized in various organelles (Figure 3).

### 2.2. CoA Degradation

Degradation of CoA-SH may be an extracellular (intestinal or systemic) or intracellular process [23]. The extent of CoA-SH degradation depends on the supply of CoA-SH in the food and physiological state of the organism, as it determines CoA-SH usage in different metabolic processes [31].

#### 2.2.1. Extracellular Degradation of CoA (Known as Intestinal or Systemic)

The CoA-SH degradation pathway is presented in Figure 2 and Figure 3. This pathway is catalyzed by the membrane or soluble enzymes, and not all of them are specific for CoA degradation. In systemic degradation they can hydrolyze not only the CoA-SH but also different types of acyl-CoA. However, it is not well known at which step of the acyl-CoA degradation process, the acyl group is removed [37]. The degradation of CoA-SH to pantetheine (PanSH) and finally to Pan, which is used as a substrate for intracellular CoA-SH synthesis, is a sequence of reactions including dephosphorylations and removal of nucleotide moiety (Figure 2). One of the first steps is the hydrolysis of diphosphate bond in CoA-SH, with the release of PPanSH, catalyzed by the alkaline ectonucleotide pyrophosphatase/phosphodiesterase (ENPP). Those are the promiscuous enzymes, which break down the 5′-phosphodiester bonds in nucleotides and their derivatives, may use not only the CoA-SH but also dPCoA as a substrate (dPCoA is preferred) [38,39].

In the intestinal degradation of CoA-SH the first step is most likely catalyzed by alkaline phosphatase (AP), an enzyme highly abundant in the intestinal lumen [25]. The product of this reaction, dPCoA, is later hydrolyzed by ENPP with the release of 5′-AMP and PPanSH. In systemic degradation, ENPP hydrolyzes the phosphodiester bond in CoA-SH, transforming CoA-SH into PPanSH and producing 3′,5′-ADP. Two ENPP isoforms (1 and 3) are probably involved in this process. Both are transmembrane glycoprotein homodimers, but some soluble forms have also been detected [40,41]. PPanSH released from CoA-SH or dPCoA is then dephosphorylated by AP or other unknown, phosphatases to PanSH.

The last step of the extracellular CoA-SH degradation pathway is catalyzed by a specific regulatory enzyme—pantetheinase, also known as vanin (VNN). Pantheteinase produces Pan and cysteamine from PanSH by specific cleavage of an amide bond, thus providing Pan as a precursor for intracellular CoA-SH synthesis [42]. Two isoforms (VNN1 and VNN3) are present in mice, and three isoforms are present in humans (VNN1-3) [43,44,45]. VNN1 is the most widely expressed GPI-anchored ectoenzyme. It is found in the membranes of epithelial cells, including ileal enterocytes and kidney proximal tubule epithelial cells. An additional soluble form secreted from hepatocytes has also been detected [46,47,48]. VNN3 is found in various tissues, and this isoform is not attached to membranes, and its expression is upregulated in the inflammatory state or during oxidative stress. Together VNN1 and VNN3 contribute to extracellular pantetheine degradation [44,49].

#### 2.2.2. Intracellular Degradation of CoA

The steps of intracellular CoA-SH degradation are similar to the extracellular process. However, there are some differences, with the greatest being in the final step, when PPanSH, and eventually PanSH, is produced. Because of the lack of VNN, Pan is not produced in the intracellular process. In the cell, CoA-SH is degraded in many organelles, such as mitochondria, peroxisomes, and lysosomes. Some steps are undertaken in the cytosol.

In the mitochondrial matrix, CoA-SH and acyl-CoA are degraded by nudix (nucleoside diphosphate linked moiety X)-type motif (NUDT8) to PPanSH or acyl-4′-P-pantetheine, respectively, with the release of 3′,5′-ADP [50]. The family of NUDT hydrolases contains enzymes with a high affinity for acyl-CoA. Along with mitochondrial NUDT8, two other NUDT hydrolases (NUDT7 and NUDT19) participate in the intracellular CoA-SH degradation process. They differ not only in subcellular localization but also in a regulation pattern, size, and tissue distribution [50,51,52]. Mitochondrial NUDT8 is widely distributed among tissues. In mice, the highest amounts of NUDT8 are found in mitochondria of kidneys, heart, brown adipose tissue, liver, brain, heart, and skeletal muscles. NUDT8 hydrolyzes CoA-SH and short- and medium-chain acyl-CoA to acyl-4′-P-pantetheine [50]. The fate of the acyl-PPanSH produced in mitochondria is currently unknown. Possibly it is degraded by ACOT, but this remains speculative since there is no evidence.

Acyl-CoA degradation in peroxisomes is similar to that in the mitochondrial pathway. NUDT7 and NUDT19 are involved. NUDT7 is active against short- and medium-chain acyl-CoAs, and this activity is higher than that against CoA-SH. In mouse liver, its expression is regulated by feeding-fasting cycles, probably through PPARα action [50,51,52,53]. NUDT19 in mice is found mostly in kidneys. Its main substrate is CoA-SH, but it also shows high activity against some short- and medium-chain acyl-CoAs [52,54]. As in mitochondria, the acyl-CoAs are degraded to acyl-4′-P-pantetheine. Based on unpublished experimental results, Hunt et al. suggest that the recombinant mouse ACOT3 and ACOT8 both hydrolyze lauryl-PPanSH [55]. Antonenkov and Hiltunen suggest that the removal of fatty acids from acyl-4′-P-pantetheine could allow the PPanSH to exit peroxisomes, and enter the cytosol through a specific transporter, or freely through the membrane [56]. However, there is no clear evidence shows that PPanSH, whether produced from free CoA or acyl-CoA, reaches the cytosol [23].

Lysosomal degradation of acyl-CoA is different from peroxisome and mitochondrion degradation (Figure 3). Under acidic conditions, lysosomal acidic phosphatase 2 (LAP2) removes the phosphate group from either CoA-SH or acyl-CoA, producing dephospho-acyl-CoA [57]. In contrast to other organelles, in lysosomes, long-chain acyl-CoAs are degraded. LAP2 cooperates with palmitoyl-protein thioesterase (PPT) [58,59,60]. This cooperation leads to the final lysosomal product, which is dPCoA. There is no evidence for the existence of a lysosomal dPCoA transporter, but some putative candidate proteins have been found [61].

Some evidence suggests that a small pool of PPanSH could be further degraded to PanSH by an unknown phosphatase. The most likely candidate seems to be pantothenate kinase 4 (PANK4), a bifunctional enzyme inactive as a kinase but with weak phosphatase activity towards PPanSH [25].

## 3. Tissue Levels and Intracellular Distribution

Total CoA levels (sum od CoA-SH and its thioesters) in various mammalian tissues span a range greater than 10-fold [36]. The total CoA level is relatively low in the cell, and CoA-SH is dynamically converted into thioesters; therefore, measuring CoA-SH level is challenging. The recently described method of Frank et al. [62] based on HPLC with absorbance or fluorescence output detector allows the determination of the total CoA-SH concentration in a faster and easier way than other methods, and it can be used with a broad linear range of samples from cultured cells or animal tissues. The liver, a very active organ critical for many synthesis activities, and the cardiac muscle, which depends on a high rate of substrate oxidation for energy, have the highest CoA-SH levels, followed by brown adipose tissue, kidney, brain, adrenal glands, and white skeletal muscle in rodents [33,36,63].

The total CoA concentration in the livers of ad libitum fed rats ranges from 87 to 434 nmol/g tissue [64,65,66]. The reported values vary significantly, probably because different assay methods were used in these studies. Comparable concentration of total CoA were found in the livers of mice 120–160 nm/g of wet tissue [33,62]. Liver total CoA levels and hepatic PanK activity are altered in response to nutritional state, insulin, glucagon, glucocorticoids, fibrate, and diabetes [67]. The rat heart contains approximately 100 nmol total CoA/g of tissue [31]. The concentrations of ~9 nM and ~0.3 nM of plasma CoA-SH and acetyl-CoA respectively, were reported in rat [64], however, considering that CoA-SH and acetyl-CoA are rapidly degraded in circulation [24] this issue needs further studies. The levels of total CoA in rat liver and heart are presented in Table 3.

In human hepatoma or breast adenocarcinoma where glycolysis is more intense than oxidative phosphorylation or the tricarboxylic acid cycle, the level of CoA-SH is significantly lower than it is in normal tissues. Regardless of whether the tumors are spontaneous or induced chemically, physically, or biologically, the CoA-SH level is reduced. Moreover, decreased levels of CoA-SH expressed in Lipmann Units were observed not only in cancerous tissue but also in other normal tissues of animals bearing malignant tumors [68]. In isolated hearts perfused only with Krebs–Henseleit buffer and glucose, 80% of total CoA is in the CoA-SH form, while in the presence of high concentrations of fatty acids, pyruvate, or ketone bodies, approximately 80% of CoA exists as acyl esters and acetyl-CoA [63].

The total CoA level and the CoA-SH:CoA thioester concentration ratio, together with the subcellular location of these forms, provide important parameters indicating the level of key metabolic reactions catalyzed by acyl-CoA synthetase, pyruvate dehydrogenase complex, and α-oxoglutarate dehydrogenase [63]. Due to the presence of large nuclear pores, the cytosolic CoA-SH concentration may equilibrate with that of the nucleus [23]. The level of total cytosolic and nuclear CoA in rat heart and liver ranges from 0.014 to 0.14 mM, whereas its concentrations in mitochondria and peroxisomes are significantly higher, from 2.26 to greater than 5 mM and 0.7 mM, respectively [23]. It is estimated that the pools of total CoA in heart and liver mitochondria represent approximately 95% and 80%, respectively, of the total cellular CoA [23]. Therefore, in the rat liver, the cytosolic CoA-SH level is estimated to be 20%, whereas in the heart, it is approximately 5%. In conclusion, the predominant CoA-SH pools are in the mitochondria in both the liver and heart.

The total CoA in the liver peroxisomes ranges from 2 to 4% of the total [23,63]. Moreover, the endoplasmic reticulum lumen may also contain acetyl-CoA that can be used to acetylate proteins residing in and moving through the endoplasmic reticulum [23,69,70]. The percentages of the total CoA in these pools remain unknown, but considering the data presented above, they are very low.

Depending on the metabolic state, the total cellular level of long-chain acyl-CoA is changing. These fluctuations are significant and may range from 5 to 160 μM [71]. Approximately one half of long-chain acyl-CoAs are detected in microsomes.

The large size and charge of the CoA-SH molecule induce its transport to subcellular compartments, which depends on specific high-affinity transporters located on organelle membranes [36]. Human mitochondria contain solute carrier family 25 member 42 (SLC25A42), a transporter that exchanges CoA (CoA-SH) for another adenine-containing substrate, including 3′,5′-ADP [72]. Moreover, SLC25A16 is likely to be a mitochondrial transporter for CoA-SH, although its activity has not been successfully reconstituted in vitro to date [23]. In mammalian peroxisomes, there are three members of ATP-binding cassette subfamily D, ABCD1-3, which mediated the transport of acyl-CoA [73,74]. Furthermore, it was demonstrated that SLC25A17 is localized in the peroxisomal membrane, and its main function is probably the transport of CoA-SH, FAD, and NAD^+^ into peroxisomes in exchange for intraperoxisomally generated PAP (adenosine 3′,5′-diphosphate), FMN, and AMP [75]. In the endoplasmic reticulum, the transporter AT-1 (acetyl-coenzyme A transporter) imports acetyl-CoA into the endoplasmic reticulum lumen in exchange for CoA-SH. Its downregulation results in widespread cell death and the induction of processes characteristic of autophagy [76].

## 4. Protein CoAlation and Other Protein Modifications Related to CoA

CoA-SH is involved in posttranslational modifications of proteins. On the one hand, it is a substrate in the phosphopantetheinylation process catalyzed by phosphopantetheinyl transferases. As a result of this reaction, 4′-phosphopantetheine is transferred from CoA-SH to serine residue of protein e.g., mitochondrial fatty acid synthase which results in an inactive apo-synthase being transformed into an active holo-synthase [77,78]. On the other hand, CoA-SH is a carrier of groups necessary for protein acetylation. The attachment of acetyl groups to the lysine residues is an important element in regulating the activity of many proteins. Acetylation affects the stability of the cytoskeleton elements and modulates the activity of several enzymes. It plays a particularly important role in regulating gene expression. In general, a high degree of histone acetylation correlates with increased transcriptional activity. The action of many different transcription factors and transcription co-regulators is modified by acetylation. These are the basic transcription factors such as TFIIB, but also proteins of specific cellular signal pathways such as p53, NF-κB or Rb [79,80]. Furthermore, CoA-SH can modify proteins by directly binding to cysteine residues [36].

Cysteine residues of proteins can form disulfides with low-molecular-weight thiols [81]. Such post-translational modifications generate different forms of proteins with various physicochemical properties and may also affect their functions and, in particular, modify their enzymatic activity [82,83]. Most organisms contain high concentrations of low-molecular-weight thiols. Glutathione (GSH) is the most abundant and ubiquitous of these compounds. Although <0.1% of all cysteines in proteins are modified by GSH binding (glutathionylation) in non-stressed cells, this portion increases to >15% in disulfide stress conditions [84]. Other important compounds from this group found in mammals include cysteine [84], homocysteine [85], and CoA-SH [36,84]. CoA-SH may covalently bind to cysteine residues in proteins, similar to glutathione, in a process called CoAlation [83,86,87,88].

Modification of cell proteins by CoAlation is stimulated in response to oxidative stress. Under stress conditions, cysteine thiol groups are oxidized to sulfenic acid, which facilitates the formation of disulfide bonds between cysteine residues and CoA-SH (and other low-molecular-mass thiols); this process may be effectively blocked by antioxidants [36,86]. It has also been shown that the level of CoAlation of proteins correlates with the concentration of CoA-SH in the cell [36]. CoAlation, similar to glutathionylation, is a reversible process, and protein de-CoAlation is a rapid event initiated immediately when oxidative stress is eliminated [86]. Although the mechanism of in vitro CoA-SH attachment is recognized as nonenzymatic, enzymes in the cell may be involved in this process. It was found that the removal of covalently attached CoA to proteins involves enzymes (CoAredoxins) [36]. In addition to oxidative stress, the increased CoAlation of proteins can also be caused by starvation in the rat liver. The opposite effect is caused by feeding rats for seven days with a high-fat and sucrose-rich diet. The CoAlation of proteins is also significantly lower in the liver of genetically obese ob/ob mice [36,86].

More than 500 proteins in mammalian cells have been identified as CoAlated, and the majority (>65%) are metabolic enzymes involved in major metabolic pathways, such as the tricarboxylic acid cycle, β-oxidation, ketone bodies, glucose and amino acid metabolism, in which CoA-SH and its derivatives are cofactors and metabolites. Among the proteins modified with CoA-SH, a significant pool consists of proteins that are also involved in stress response and protein synthesis [36,86].

Cysteine residues modified by CoA-SH are often located in protein sites that are functionally and structurally important and that are involved in many different biological functions, such as enzymatic catalysis, signal transduction, structure stabilization, and metal binding [36]. Below are some examples of proteins that undergo the devastation and the effects of this modification on their function. CoAlation of rat mitochondrial acetyl-CoA acetyltransferase transformed this enzyme into two partially active forms, with approximately 70% and 50% of the activity of the unmodified enzyme [82,89,90]. Under severe oxidative stress, the antioxidant enzymes, 2-Cys peroxiredoxin subclass Prdx5 are also CoAlated and lose their activity as a result [91]. Under oxidative stress, an increase in the CoAlation of pyruvate dehydrogenase kinase 2 (PDK2) in heart caused a decrease of its activity that leads to dephosphorylation of the pyruvate dehydrogenase (PDH) in pyruvate dehydrogenase complex (PDC) and increase of its activity [86]. The activity of Aurora A protein kinase, an enzyme overexpressed in various cancers, decreases upon CoAlation, and the inhibition of this kinase may be a new potential direction for the development of anticancer therapy [92].

In summary, CoAlation is a reversible process that is assumed to protect the cysteine residues in proteins from irreversible oxidation. It also regulates the activity of certain proteins whose cysteine residues are essential for their functioning. Among proteins susceptible to CoAlation stimulated by oxidative or metabolic stress, a high proportion of metabolic enzymes may represent the potential regulatory mechanism by which these functional pathways react to changes in the CoA/acyl-CoA ratio in the cell [86].

## 5. CoA and Pathologies

Diseases related to improper cellular concentrations of CoA-SH are not very common. This outcome is a result of Pan, being prevalent in most foods, and even ruminant microbiota produce enough Pan to adequately supply human organisms [31]. Thus, diseases related to improper CoA-SH levels are generally related to defects in the genes associated with CoA-SH synthesis, CoA-SH degradation or transport [93]. However, one paper described the inverse correlation between Pan intake and serum C-reactive protein concentration in human subjects and suggested that this correlation was associated with CoA-SH levels, but in this study, molecular level experiments were not performed [94]. Nevertheless, there are some well-characterized pathologies related to CoA-SH concentration.

### 5.1. Neurodegenerative Diseases

Perhaps the best described disease associated with reduced cellular CoA-SH levels is pantothenate kinase-associated neurodegeneration (PKAN), also known as Hallervorden–Spatz disease, an autosomal recessive inherited disease caused by a mutation in the *PANK2* gene. It was first described in 1922 by two physicians from Germany, Hallervorden and Spatz. This disease is characterized by neurodegeneration, iron accumulation in the brain, and variable neurological dysfunction. It is caused by specific mutation in the *PANK2* gene that results in deficiency of the PANK2 enzyme, which leads to the accumulation of cysteine and the chelation of iron in the brain [95]. *In vitro* and animal studies confirmed that this disease is also associated with a decrease in CoA-SH and CoA thioesters in brain and liver cells [78,96]. The roles of CoA-SH levels in the development of PKAN have been presented in two recent papers. Lambrechts et al. [78] suggested that reduced CoA-SH biosynthesis leads to a reduction in active mitochondrial acyl carrier protein and the reduced lipoylation and activity of PDC. These findings were confirmed based on similar clinical features (e.g., progressive dystonia) of three other genetic diseases. Mutation of gene encoding another enzyme of the CoA-SH synthesis pathway, *COASY*, leads to CoA synthase protein-associated neurodegeneration (CoPAN), which is associated with partial loss of COASY activity. This disease has very similar clinical features to PKAN, including iron accumulation in the brain [97]. Two other CoA-related neurodegenerative diseases are not associated with CoA-SH synthesis: MePAN, which is caused by mutation of mitochondrial enoyl-[acyl carrier protein] reductase, a protein critical for lipoylation, and dihydrolipoyl transacetylase (PDC-E2) deficiency. All these pathologies result in decreased PDC activity [78]. Results from a study using a PKAN mouse model suggested another pathological mechanism of a disease that involves reduced succinyl-CoA levels and reduced expression of genes associated with heme and hemoglobin synthesis, which may lead to disruption of oxygen availability and/or redox balance in the brain [96]. CoA-SH supplementation prevented neuronal death in vitro suggesting that CoA-SH treatment may be a possible therapeutic intervention for PKAN patients [98]. Recently, a new potential therapeutic approach for PKAN treatment was reported. An allosteric PANK activator from the pantazine group, PZ-2891, has been successfully used for PKAN treatment in a mouse model [99].

Variants of the *COASY* gene with near complete loss of function cause a subtype of pontocerebellar hypoplasia (PCH) [100]. PCH is a heterogeneous group of neurodegenerative diseases. Its clinical features include hypoplasia of the cerebellum and pons. Patients with biallelic *COASY* mutations develop prenatal PCH and die before or shortly after birth. In these patients, no stable COASY was produced in cells. Unfortunately, the CoA-SH levels were not measured in this study, but it is generally agreed that the synthesis of CoA-SH from Pan is the only source of CoA in the human body [100].

The risk of Alzheimer’s disease (AD) development has been associated with single nucleotide polymorphisms (SNPs) in the *COASY* gene [101]. However, this study focused only on women with Down syndrome; therefore, this finding needs to be confirmed in the general population. Moreover, AD is associated with increased methylation of the *COASY* gene, which may lead to its decreased expression [102]. Cholinergic dysfunction in AD is caused by the reduced production of acetyl-CoA [103]. All these data suggest that alterations in CoA-SH synthesis may be involved in the development of AD.

Another neurodegenerative disorder, Huntington’s disease (HD), seems to be associated with CoA-SH levels. Specifically, a post-mortem metabolomic study revealed decreased levels of Pan in the brains of HD patients [104]. Decreased Pan in the brain may cause myelin metabolism disorders that can lead to myelin loss in HD patients [6].

SLC25A42 is a CoA-SH transporter located in the inner mitochondrial membrane. Recently, two papers reported disease caused by mutations in the gene encoding SLC25A42 in Arab patients [105,106]. This disease presented with epileptic encephalopathy, developmental delay, myopathy and lactic acidosis. The intracellular total CoA level in fibroblasts isolated from these patients was decreased [105].

### 5.2. Cancers

Carcinogenesis is inextricably associated with substantial changes in cell metabolism. There is a significant amount of research indicating that changes to carbohydrate, nucleic acid, lipid, and amino acid metabolism are critical in the development, progression, and metastasis of many types of cancers [19,107,108]. There is also growing evidence that pathways of CoA-SH biosynthesis can undergo severe changes in cancer cells. However, most of the research is focused on genetic level data, and few mention CoA-SH level.

Some research has shown that increased levels of CoA-SH are toxic to cancer cells, including breast, cervical, and liver cancer cells. Cell cultures showed inhibited growth after 24 h of incubation with 100 µM CoA-SH. Unfortunately, at the same time- and dose-range, CoA-SH was found to be equally toxic to normal cells; therefore, the potential of using CoA-SH as a therapeutic agent is limited [109]. On the other hand, a decrease in the level of CoA-SH can be tolerated very well by cancer cells. Lowering the level of CoA-SH by 50% did not change cell viability; however, notably, these cells were monitored for only 8 days in this study [110].

The abnormal biosynthesis and homeostasis of CoA-SH can lead to dysregulated CoAlation of Aurora A kinase. This kinase controls meiosis, mitosis, and cell division, thus, it is associated with various types of cancer. The overexpression of Aurora A was detected in leukemia, breast, prostate, and colon cancers [111]. It has been shown that CoA-SH binds directly to Aurora A and inhibits its catalytic activity in a dose-dependent manner [92]. The link between CoA-SH level and Aurora A action suggests interesting possibilities for a new therapeutic approach.

Acute myeloid leukemia (AML) is characterized by broad heterogeneous genetic alterations. Recently, two new genetic markers of AML in CoA-SH biosynthesis pathways were proposed: *PANK2* and *PANK4*. Overexpressed *PANK2* was a favorable prognostic factor, while high expression of *PANK4* was associated with a low survival rate [112]. The possible mechanisms behind these effects are still unclear and require further investigation. It has been proven that PANK4 can inhibit pro-caspase-9 expression; therefore, it is possible that, by suppressing cell apoptosis, PANK4 leads to shorter survival of patients with AML [113]. Of these two genes, only the proteins encoded by *PANK2* have functional pantothenate kinase activity; however, researchers did not analyze the CoA-SH level in tissues or serum [31]. Furthermore, a *VNN1* gene was proposed for improving therapy outcomes and as relapse risk biomarker. Higher expression of *VNN1* was associated with poor outcomes, including resistance to chemotherapy and shorter relapse periods [114].

On the molecular level, breast cancer is divided into subtypes based on the expression level of estrogen receptor (ER), progesterone receptor (PR), and human epidermal growth factor receptor 2 (HER2). An increasing number of studies have investigated the genomic and transcriptomic architectures of these subtypes, but few incorporated CoA-SH biosynthesis genes into the analyses. Nagai et al. discovered a significantly higher expression of *PANK3* in ER+/PR+ primary breast tumors than in ER-/PR- samples [115]. Unfortunately, no other types of breast cancer were analyzed, and no comparison to normal tissue was performed. It is also unclear whether a higher expression of *PANK3* is correlated with a higher CoA-SH level. The data from in vitro experiments suggested that *PANK2* is downregulated by estrogen receptor β; however, this observation has not been confirmed in human tissues [116]. The expression pattern of *COASY* in breast cancer cells was not compared to that in normal cells; however, some studies have reported that knocking out *COASY* in triple-negative breast cancer (ER-/PR-/HER2-) resulted in reduced cell proliferation and migration. Interestingly, the effect was achieved only in RNAi-knockdown models, and knocking down *COASY* with inducible shRNA had no effect on cell proliferation [110]. Furthermore, knocking down *COASY* by siRNA sensitized HER2+ (ER±/PR±/HER+) breast cancer cells to trastuzumab treatment [117]; however, this effect needs to be verified in in vivo models. Standard chemotherapy for breast cancer, regardless of ER/PR.HER2 status damages the ovaries, causing temporary or permanent chemotherapy-related amenorrhea (CRA) in many women who survive cancer [118]. Unfortunately, the currently available methods for predicting preservation of ovarian function after cancer treatment are inadequate [119]. Recently, a novel genetic predictor of CRA based on CoA metabolism was proposed. Specific SNPs of PPCDC can be related to lower chances of menses after chemotherapy [120].

Colorectal cancer is one of the most common cancers and one of the most common causes of cancer-related death worldwide [121]. Although radiation and neoadjuvant concurrent chemoradiotherapy are currently the gold standard therapies, no validated biomarkers or molecular targets to predict or improve patient response have been identified. Recently, COASY was proposed as a predictive marker for radiation resistance. *In vitro* and *in vivo* studies proved that increased levels of COASY were directly related to radiation resistance, possible by enhancing DNA repair efficiency and/or by activation of the PI3K/AKT/mTOR pathway. Knocking down *COASY* resulted in a better radiation response, including delayed tumor growth, inhibited cell proliferation, and increased activation of apoptosis after irradiation [122,123]. It would be interesting to know whether heterogeneity in response to radiation alterations in *COASY* expression levels is characteristic of other types of cancers. Another gene from the CoA-SH metabolism pathway, *VNN1*, was proposed as a marker for the response to preoperative neoadjuvant concurrent chemoradiotherapy (CCRT). Studies have shown that high expression of *VNN1* is associated with poor response to CCRT and an adverse prognosis [124]. However, VNN1 promotion of tumor progression seems to be more closely related to oxidative stress conditions than to CoA-SH metabolism [125]. Moreover, *VNN1* and six other genes were proposed for inclusion in a blood-based biomarker panel for colorectal cancer risk assessment, which showed a very high rate of accuracy, sensitivity, and specificity [126,127].

In CoA-SH biosynthesis genes, noncoding RNAs can play roles in cancer. A microRNA-107 (miR-107) of intron 5 of *PANK1* is a posttranscriptional regulator of genes involved in adipogenesis, hypoxia, cell cycle arrest, and angiogenesis; therefore, its dysregulation may be a key factor underlying tumorigenesis. The expression level of miR-107 was found to be reduced in non-small cell lung cancer (NSCLC) and oesophageal cancer [128,129]. In contrast, miR-107 was overexpressed in pancreatic cancer and breast cancer [130,131]. *In vitro* studies suggest that miR-107, by targeting cyclin-dependent kinase 8 (CDK8), can regulate chemosensitivity to cisplatin and can be used to predict a patient’s response to chemotherapy [132].

### 5.3. Colitis

Recently, Roedriger re-examined pig experiments published in 1943 [133,134]. Pigs may have Pan deficiency, and therefore, feeding pigs a diet free of Pan resulted in the induction of colitis [134]. In humans, Pan deficiency is not found, but it is possible that lower levels of CoA-SH may be associated with the development of colitis. Some experimental data support this supposition. First, colonocyte energy metabolism is highly dependent on butyrate β-oxidation, for which CoA-SH is needed. Second, bacterial metabolites, nitric oxide and hydrogen sulfide, can lower the concentration of CoA-SH in colon mucosa [133]. SMVT encoded by the *SLC5A6* gene is critical for the intestinal absorption of biotin and Pan. Mice with the *SLC5A6* gene knocked out developed colitis. However, supplementation with high doses of biotin and Pan reversed this effect [27]. Another study suggested roles for CoA-SH levels in the development of colitis, but unfortunately, the authors did not measure the concentrations of CoA-SH in the experimental mice. In the colon tissue of patients with inflammatory bowel disease (IBD), VNN1, an enzyme of the exogenous CoA-SH degradation pathway, is overexpressed [135]. Moreover, this study showed that 3 SNPs of *VNN1* are associated with the risk of IBD. However, the association of *VNN1* expression and IBD may not be directly related to CoA-SH levels but may relate to the production of cysteamine, which is involved in the regulation of inflammation in the gut [135].

### 5.4. Myopathies

It seems that CoA-SH plays a very important role in muscle metabolism. First, it is required for the activation of fatty acids, which are energy sources for muscle, because fatty acids undergo β-oxidation. Furthermore, malonyl-CoA serves as the regulator of fatty acid transport in the mitochondria, which is a rate-limiting step of fatty acid oxidation. It was shown that the plasma concentration of Pan rises with exercise [136]. However, data on the role of CoA-SH concentrations in muscle are very scarce. Overexpressed human *PANK2* gene in mice, which resulted in increased total CoA levels in muscle, led to reduced muscle performance and strength [137]. This outcome was associated with increased oxidative stress. Therefore, this study suggests that excessively increased total CoA levels in muscle may be detrimental to muscle function [137]. However, human subjects with mutations in the gene encoding the mitochondrial transporter of CoA-SH, SLC25A42 presented with myopathy [23]. Thus, CoA-SH levels that are too low in mitochondria and CoA-SH synthesis that produces excessive CoA-SH levels both seem to be detrimental to muscle function. Moreover, it was hypothesized that the dilated cardiomyopathy and skeletal myopathy observed in Barth Syndrome (BTHS) are connected with the compromised tissue supply of CoA-SH, and treatment with Pan has been proposed. However, except for one case study from the 1990s, no publications have confirmed the benefits of Pan [138]. Rugulotto et al. [139] tried to replicate the experiment, but for three BTHS patients, this therapy was not as beneficial as was previously claimed. Thus, the treatment of BTHS patients with long-term Pan supplementation remains controversial.

### 5.5. Infectious Diseases

CoA-SH synthesis is also important for the survival and/or growth of numerous organisms that infect humans, including parasites, fungi, and bacteria.

Early studies have shown that in erythrocytes infected with malaria parasites, the level of CoA-SH is higher than in normal erythrocytes [140]. Further studies have proven that human malaria parasites can independently synthesize CoA-SH but require an exogenous supply of Pan for survival [141,142,143]. These findings resulted in the development of antiplasmodial Pan analogs that kill the parasite by depriving it of CoA-SH or inhibiting CoA-utilizing metabolic processes [144,145,146]. However, recent studies have shown that certain types of mutations in pantothenate kinase of *Plasmodium falciparum* resulted in diverse sensitivity for Pan analogs, from hypersensitive to resistant [145]. This suggests that CoA-SH metabolism can be used as a potential target of antimalarial drugs in combination therapies.

In terms of malaria, host CoA-SH metabolism is also important. Studies have shown that the levels of activity and/or expression of pantetheinase (an enzyme in the CoA-SH degradation pathway that produces Pan and cysteamine from pantetheine, encoded by *VNN* genes, see Figure 2) contribute to the susceptibility and severity of malaria [23]. Mouse strains with *VNN* mRNA expression and pantetheinase protein activity in tissues at undetectable levels showed higher parasitemia compared to their wild type counterparts [147]. Lower activity of pantetheinase leads to lower levels of anti-plasmodial cysteamine [148]. Moreover, a reduced level of serum pantetheinase activity predisposes patients to severe and complicated forms of malaria, including cerebral malaria and severe anemia, due to the diminished half-life of erythrocytes [49]. These findings seem to argue that the parasite depends on the host supply of Pan; therefore, a lower level of Pan would not be expected to benefit to *Plasmodium*. However, researchers have speculated that the effects of pantetheinase activity on the growth of the malaria-causing parasite might vary at different stages of infection. Unfortunately, none of these studies assessed how changes in enzyme activity from the degradation pathway of CoA-SH influence CoA-SH levels.

In animal models, pantetheinase deficiency reduced the negative impact of inflammation resulting from pathogen infection on host fitness. In two models, schistosomiasis and rickettsiosis, undetectable levels of *VNN* expression resulted in increased survival and tolerance for the disease [149]. After *Schistosoma* infection, mice with *VNN* knocked out showed better controlled inflammatory reactions and attenuated intestinal injury [150]. In the case of *Rickettsia* infection, *VNN*-knockout mice exhibited decreased formation of granulomas in the spleen and liver [151].

An increasing number of multidrug-resistant bacterial strains have created an emerging need for the development of novel antibiotic strategies, and CoA metabolism can be a novel antimicrobial target. During recent decades, a number of studies have reported that pantothenamides (analogs of Pan) possess activity against gram-positive and gram-negative bacteria, including *Escherichia coli*, *Staphylococcus aureus*, methicillin-resistant *S. aureus* (MRSA), *Staphylococcus epidermidis*, *Streptococcus pneumoniae*, *Streptococcus pyogenes*, *Pseudomonas aeruginosa*, *Mycobacterium avium*, *Mycobacterium abscessus*, and *Mycobacterium kansasii*. Pantothenamides can act as inhibitors (either competitive or allosteric) of pantothenate kinase [152,153,154,155], causing cellular depletion of CoA-SH, or as substrates for pantothenate kinase, creating CoA analogs that disrupt CoA-dependent reactions (e.g., bacterial fatty acid biosynthesis) [156,157,158]. Nonetheless, these compounds have never been tested in vivo due to their instability in serum. It became apparent that human serum pantetheinase, an enzyme that normally degrades pantetheine, can also hydrolyze pantothenamides. Researchers are trying to overcome this problem with two different approaches: one is based on developing stable pantothenamides that are pantetheinase-resistant, and the other is based on using a combination of pantothenamides and pantetheinase inhibitors [154,159]. Both strategies have shown promising results in in vitro testing.

### 5.6. Diabetes

Studies have shown that total CoA levels in the liver and heart of diabetic rats and mice are higher than those in healthy rats and mice [160,161]. Moreover, in ob/ob mice (well-established models of human type 2 diabetes mellitus (DM2)), PANK1 deficiency leads to reduced serum insulin, improved insulin tolerance, and reduced fasting blood glucose [162].

MicroRNAs have been reported to play important roles in the pathophysiology of DM2 [163,164]. Studies have shown that microRNA-103 (miR-103) and miR-107, both located in PANK genes, play important roles in insulin sensitivity. In ob/ob mice, the expression of miR-103 and miR-107 in liver and adipose tissue is elevated. Silencing miR-107/103 in the liver resulted in improved glucose homeostasis and insulin sensitivity, whereas silencing miR107/103 in adipocytes resulted in upregulated expression of caveolin-1, which activates insulin signaling, probably by stabilizing caveolae-associated insulin receptors [165,166]. Moreover, circulating miR-103 has been recently proposed as an early marker of prediabetes mellitus stage [167].

### 5.7. Other Diseases

In the majority of cases, the underlying pathogenic mechanisms that lead to autism spectrum disorders are complex and not very well defined. There is growing evidence that gene dosage changes (changes in the number of copies of a particular gene) can contribute to the etiology of autism. Some of the copy number variants described in patients with autism are related to an alteration in the *PPCDC* gene [168]. Changes in the copy number of the *PPCDC* gene can result in mitochondrial defects that can affect synaptic function and result in neuropsychiatric symptoms [169].

In experiments, obese mice in the fed state had twice the total CoA levels in the liver and skeletal muscle than their nonobese littermates. Moreover, during fasting, an increase in total CoA level was observed only in the nonobese group, [170]. Of all *PANK* genes, only the expression of *PANK1* was upregulated in the liver of the obese mice.

## 6. Single-Nucleotide Polymorphisms in Genes Involved in CoA Metabolism

Genes involved in CoA-SH biosynthesis and degradation pathways seem to exert a variable effect on human traits, including under pathological conditions. Genome-wide association studies (GWAS) have provided great opportunities to discover relationships between SNPs in genes and various metabolic phenotypes. However, in the case of CoA-SH biosynthesis and degradation pathways, much remains unknown. Only a few genes in the CoA-SH biosynthesis and degradation pathway have shown some associations with biological traits (Table 4). Moreover, according to the GWAS catalogue (an online database of SNP-trait associations), no associations have been discovered to date between SNPs in genes not involved directly in CoA metabolism and CoA-SH levels [171]. This lack of information suggests a new research opportunity.

## 7. CoA and Its Precursor Pantethine as Circulating Lipid-Lowering Supplemental Agents

Since CoA-SH plays a key role in lipid metabolism, especially in fatty acid oxidation, scientists have hypothesized that CoA-SH or its precursor supplementation might reduce circulating lipid concentration. Indeed, it has been reported that the combination of CoA-SH (used at pharmacological doses) with a moderate dose of a statin was more effective in curing patients with mixed dyslipidemia than statin monotherapy [184]. Moreover, it has been shown that combined CoA-SH and statin therapy was more effective in improving triacylglycerol, total cholesterol, and non-HDL-cholesterol concentrations than statin alone in patients with metabolic syndrome and mixed hyperlipidemia [185].

Much more attention has been given to pantethine (a CoA-SH precursor) as a circulating lipid-lowering compound than to CoA-SH. Pantethine, a stable form of pantetheine (two molecules of pantetheine linked by a disulfide bridge) as a dietary supplement (used at a pharmacological dose of 600–1200 mg per day), lowers elevated levels of total cholesterol, LDL-cholesterol, triacylglycerol, and non-HDL-cholesterol concentrations [186,187,188]. The effect of orally administered pantethine also results in: (a) an increase in HDL cholesterol concentration; and (b) normalization of apolipoprotein B (apo B) and apolipoprotein A (apoA); however, the effect depends on the type of dyslipidemia [189,190,191].

Some data suggest that CoA-SH (used at 400 U per day) can improve blood triacylglycerol and lipoprotein concentrations (total cholesterol and non-HDL cholesterol were significantly reduced, and HDL cholesterol was increased) to a greater extent than pantethine (used at 600 U per day) [192].

Taken together, the results published thus far indicate that CoA-SH and pantethine can be useful in lowering elevated levels of circulating lipids in some diseases. This effect is most impressive when pantethine side effect and toxicity (practically none, when used at concentrations effectively lowering blood lipid concentration) are compared with commonly used drugs that lower circulating lipids (for instance, statins). However, the effect of CoA-SH or pantethine on circulating lipids is relatively slow. Usually, a maximal effect is observed at 4 months but may take up as long as 6–9 months.

The exact molecular mechanism of action of CoA-SH and pantethine on blood lipid concentration is unknown. Although pantethine is a precursor for vitamin B5 synthesis and intake of the pharmacological dose of pantethine results in higher circulating vitamin B5 concentration, the production of vitamin B5 is not the mechanism of action because intake of panthotenic acid does not have the same effect on serum lipid concentration [2]. Pantethine-induced inhibition of acetyl-CoA carboxylase by cysteamine, the product of pantethine and CoA degradation, and inhibition of HMG-CoA reductase and cholesterol synthesis in isolated hepatocytes by pantethine may explain, at least in part, the fact that pantethine (and possibly CoA-SH) administration in pharmacological doses is effective in reducing plasma triacylglycerol and cholesterol (total cholesterol, LDL-cholesterol, and non-HDL-cholesterol) concentrations [193,194,195].

Pantethine (specifically, the cysteamine formed from pantethine) inhibition of acetyl-CoA carboxylase decreases the level of malonyl-CoA, which is (a) a substrate for fatty acid synthesis; and (b) an allosteric inhibitor of CPT1, a key regulator of fatty acid oxidation. Consequently, this reduction of malonyl-CoA leads to (a) a decrease in fatty acid synthesis; and (b) an increase in fatty acid oxidation in mitochondria. In turn, plasma lipids are affected, especially by triacylglycerol-lowering effects.

However, another mechanism is not excluded. Considering that (a) gut microbiota (especially bacterial strains such *Lactobacillus* and *Bifidobacterium*) aid in decreasing lipids in hyperlipidemic patients; and (b) pantethine promotes the survival and growth of various beneficial gut bacteria; it has been suggested that microbiota can contribute (at least in part) to a possible mechanism of pantethine action on circulating lipids [188,194,195,196,197,198,199].

**Figure 4:**
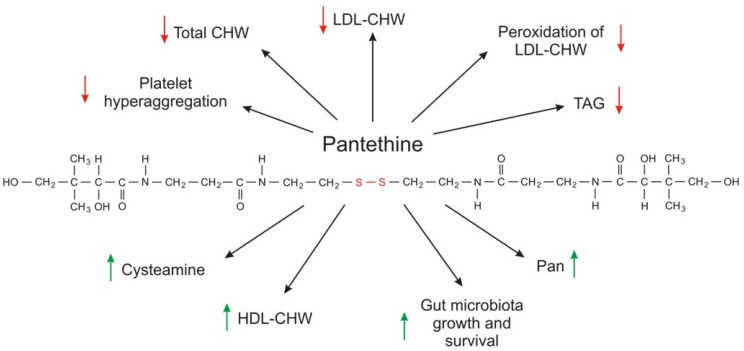
The metabolic effects of pantethine. Red arrows indicate decrease; green arrows indicate increase.

Pantethine supplementation also has some beneficial effects on parameters associated with platelet lipid composition and cell membrane fluidity. In diabetic patients, the lipid composition of platelets is significantly different than that of healthy subjects. Supplementation with pantethine normalizes platelet fatty acid composition to a control value, leading to a significant reduction in platelet hyperaggregation [200]. Moreover, pantethine inhibits lipid peroxidation of the LDL-cholesterol fraction and consequently reduces lipid deposition, intimal thickening, and fatty streak formation in the aorta and coronary artery [201]. The metabolic effects of pantethine are summarized in Figure 4.

Although several clinical trials have shown that CoA-SH and especially pantethine used at pharmacological doses reduce circulating lipid levels in patients with dyslipidemia associated with different pathologies, it seems that additional studies are necessary to determine whether CoA-SH or pantethine supplementation has a beneficial effect on cardiovascular risk markers independently of or in combination with a healthy diet. Moreover, further research is also needed to explain the exact molecular mechanism of CoA-SH and pantethine action on circulating lipid concentration. At present, it is safe to say that administration of CoA-SH is not entirely different from the administration of pantethine because in the end both compounds are converted to Pan and cysteamine.

## 8. Conclusions and Further Perspectives

The key roles of CoA-SH in cell metabolism have been well documented. However, further studies are required to address the following areas of interest: (a) the significance of changes in CoA-SH levels under pathological conditions, including neurodegenerative diseases, cancers, colitis, myopathies, infectious diseases, diabetes; and (b) the potential role of CoA-SH and pantethine as dietary supplements lowering circulating lipids.

## Figures and Tables

**Table 1:** Examples of reactions with the participation of CoA-SH as a substrate in human cells; based on Ridgway and Mcleod and the UniProt database [7,13].

CoA-SH as a Substrate			
	Enzyme	Reaction	Process
Lipid metabolism	acyl-CoA synthetases (ACS)	fatty acid + **CoA-SH** + ATP → fatty acyl-CoA + AMP + PP_i_	fatty acids activation
	carnitine palmitoyltransferase 2 (CPT2)	acylcarnitine + **CoA-SH** → carnitine + fatty acyl-CoA	carnitine shuttle
	thiolases e.g., β-ketoacyl-CoA thiolase	acyl-CoA + **CoA-SH** → acyl_(n carbon-2)_-CoA + acetyl-CoA acetoacetyl-CoA + **CoA-SH** → 2 acetyl-CoA	fatty acids oxidation ketone bodies oxidation
	ATP-citrate lyase (ACLY)	citrate + ATP + **CoA-SH** → *oxaloacetate* + acetyl-CoA + ADP + P_i_	lipogenesis, synthesis of cholesterol and others
Carbohydrate metabolism	pyruvate dehydrogenase complex (PDC)	pyruvate + **CoA-SH** + NAD^+^ → acetyl-CoA + NADH + H^+^ + CO_2_	oxidative decarboxylation of pyruvate
Amino acids metabolism	branched-chain α-keto acid dehydrogenase complex	α-ketoisovaleric acid + **CoA-SH** + NAD^+^ → isobutyryl-CoA + NADH + H^+^ + CO_2_ α-ketoisocapronic acid + **CoA-SH** + NAD^+^ → iso-valeryl-CoA + NADH + H^+^ + CO_2_ α-keto-β-methylvaleric acid + **CoA-SH** + NAD^+^ → α-methylbutyryl-CoA + NADH + H^+^ + CO_2_	oxidative decarboxylation of branched-chain α-keto acids
Lipid, carbohydrate, amino acids and ethanol metabolism	α-oxoglutarate dehydrogenase complex	α-oxoglutarate + **CoA-SH** + NAD^+^ → succinyl-CoA + NADH + H^+^ + CO_2_	tricarboxylic acid cycle
	acetyl-CoA synthetase	acetate + **CoA-SH** +ATP → acetyl-CoA +AMP + PP_i_	ethanol metabolism, acetate formed by gut microbiota metabolism

**Table 2:** Examples of reactions involving CoA-SH as a product in human cells; based on Ridgway and Mcleod and the UniProt database [7,13].

CoA-SH as a Product			
	Enzyme	Reaction	Process
Lipid metabolism	fatty acid synthase (FASN)	7 malonyl-CoA + acetyl-CoA + 14 NADPH + 14 H^+^ → palmitate + 14 NADP^+^ + 7 CO_2_ + 6 H_2_O + **8 CoA-SH**	lipogenesis
	fatty acid elongases (ELOVLs)	fatty acyl-CoA + malonyl-CoA → β-keto-acyl-CoA + CO_2_ + **CoA-SH** or fatty acyl-CoA + acetyl-CoA → β-keto-acyl-CoA + **CoA-SH**	microsomal elongation of fatty acid chains mitochondrial elongation of fatty acid chains
	acyltransferases e.g., diacylglycerol O-acyltransferase (DGAT) e.g., acyl-CoA:cholesterol acyltransferase (ACAT)	1,2-diacylglycerol + fatty acyl-CoA → triacylglycerol + **CoA-SH** cholesterol + acyl-CoA → cholesteryl ester + **CoA-SH**	triacylglycerol synthesis cholesterol metabolism
	carnitine palmitoyltransferase 1 (CPT1)	carnitine + acyl-CoA → acylcarnitine + **CoA-SH**	carnitine shuttle
	3-hydroxy-3-methylglutaryl-CoA reductase (HMGR)	HMG-CoA + 2 NADPH + 2 H^+^ → mevalonate +2 NADP^+^ + **CoA-SH**	synthesis of cholesterol, cholecalciferol (skin), prenyl moieties
	acyl-CoA thioesterases	fatty acyl-CoA + H_2_O → free fatty acid + **CoA-SH**	regulation of intracellular levels of acyl-CoA, free fatty acids and CoASH
Lipid, carbohydrate, amino acids and ethanol metabolism	citrate synthase	acetyl-CoA + oxaloacetate + H_2_O → citrate + CoA-SH	tricarboxylic acid cycle
	succinate thiokinase (also called succinyl-CoA synthetase)	succinyl-CoA + ADP (GDP) + P_i_ → succinate + ATP (GTP) + **CoA-SH**	tricarboxylic acid cycle
Others	acetyltransferases e.g., choline O-acetyltransferase e.g., histone acetyltransferase (HAT)	choline + acetyl-CoA → acetylcholine + **CoA-SH** histone-Lys + acetyl-CoA→ histone-Lys-acetyl + **CoA-SH**	neurotransmitters synthesis protein acetylation

**Table 3:** The level of total CoA in different tissues of the rats [23,31,64,65,66].

Tissue			Total CoA Concentration/Level
Liver			87–434 nmol/g tissue
	Subcellular compartment	cytosol	0.1–0.14 mM
		mitochondria	5.29 mM
		peroxisomes	0.7 mM
Heart			~100 nmol/g tissue
	Subcellular compartment	cytosol	0.014 mM
		mitochondria	2.26 mM

**Table 4:** Traits associated with genetic variants of CoA biosynthesis and degradation enzymes. IGFBP3—insulin-like growth factor-binding protein 3, BMI—body mass index, AD—Alzheimer’s disease, and IBD—inflammatory bowel disease.

Enzyme	Gene	SNP Variant	Associated Trait	Nature of Change	Tested Population	Reference
Pantothenate kinase	*PANK1*	rs11185790-A	Insulin level	Decreased insulin level	European	[172]
		rs7073802-A	Educational attainments	Increased self-reported math ability	European	[173]
	*PANK3*	rs35693458-A	Unipolar depression	Increased probability of major depressive disorder in individuals not exposed to trauma	European	[174]
	*PANK4*	rs12073504-G	Obesity-related trait	Increased IGFBP3	Latin American	[175]
		rs7535528-G	BMI	Increased BMI	East Asian, African American, European, South Asian, Latin American	[176]
		rs7535528-A	BMI	Decreased BMI	European	[177]
		rs7535528-A	Neuroticism	Increased irritability	European	[178]
Phosphopantothenoylcysteine decarboxylase	*PPCDC*	rs2120019-C	Blood trace element	Decreased serum Zn levels	European	[179]
		rs12148488-T rs8042558-T	Coffee consumption	Decreased consumption	European	[180,181]
		rs147451859-G	Response to chemotherapy	Adverse response to antineoplastic agent in breast cancer	European	[120]
		rs12148488-G	Blood pressure	Decreased mean arterial pressure	African American, Latin American, European	[182]
Coenzyme A synthase	*COASY*	rs668799-T	Medication use	Increased drugs used in diabetes	European	[183]
		rs598126-T	AD	Increased risk of AD	American	[101]
Pantetheinase	*VNN1*	rs3756975-C rs13204527-T rs909977-T	IBD	Increased risk of IBD	European	[135]

## Abbreviations

ABCD	ATP-binding cassette subfamily D
ACAT	Acyl-CoA:cholesterol acyltransferase
ACBP	acyl-CoA binding protein
ACOT	Acyl-CoA thioesterase
ACLY	ATP-citrate lyase
ACP	Acyl carrier protein
ACS	Acyl-CoA synthetase
AD	Alzheimer’s Disease
AML	Acute myeloid leukemia
AP	Alkaline phosphate
apoA	Apolipiprotein A
apoB	Apolipoprotein B
BMI	Body mass index
CDK8	Cyclin dependent kinase 8
CoA	Coenzyme A
COASY	Coenzyme A synthase
CoPAN	CoA synthase protein-associated neurodegeneration
CPT1	Carnitine palmitoyltransferase 1
CPT2	Carnitine palmitoyltransferase 2
DPCK	Dephospho-CoA kinase
dPCoA	Dephospho-CoA
ELOVL	Fatty acid elongase
ENPP	Ectonucleotide pyrophosphatases/phosphodiesterases
ER	Estrogen receptor
FASN	Fatty acid synthase
GSH	Glutathione
GWAS	Genome-wide association studies
HD	Huntington’s Disease
HER2	Human epidermal growth factor receptor
HMG-CoA	3-hydroxy-3-methylglutaryl-CoA
HMGR	3-hydroxy-3-methylglutaryl-CoA reductase
IBD	Inflammatory bowel disease
IGFBP3	Insulin-like growth factor-binding protein
IMM	Inner mitochondrial membrane
LAP	Lysosomal acid phosphatase
NSCLC	Non-small cell lung cancer
NUDT	Nucleoside diphosphate linked moiety X-type motif
OMM	Outer mitochondrial membrane
Pan	Pantothenate, pantothenic acid
PANK	Pantothenate kinase
PCH	Pontocerebellar hypoplasia
PDC	Pyruvate dehydrogenase complex
PDK	Pyruvate dehydrogenase kinase
PKAN	Pantothenate Kinase Associated Neurodegeneration
PPan	4′phosphopantothenate
PPanSH	4′-phosphopantetheine
PPCDC	Phosphopantothenoylcysteine decarboxylase
PPCS	Phosphopantothenoylcysteine synthetase
PPT	Palmitoyl-protein thioesterase
PR	Progesterone receptor
ROS	Reactive oxygen species
SLC25A42	Solute Carrier Family 25 Member 42
SMVT	Sodium dependent multivitamin transporter
SNP	Single nucleotide polymorphisms
VNN	Pantetheinase
